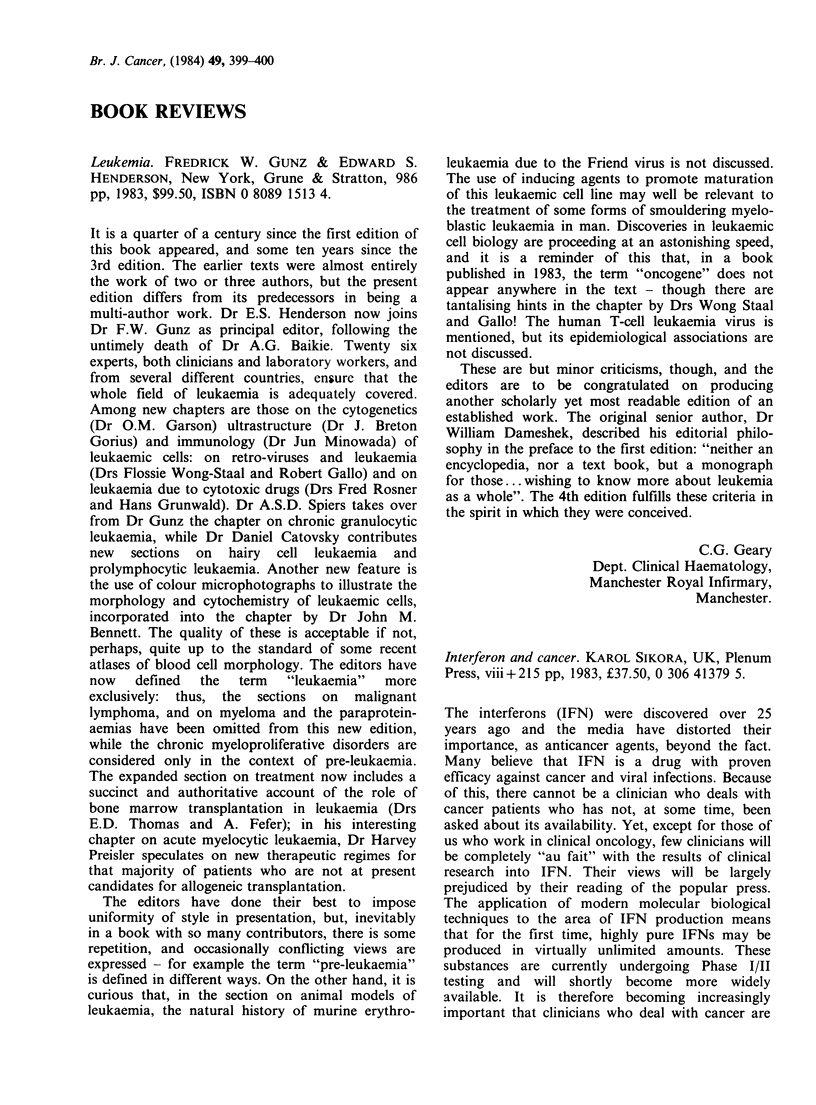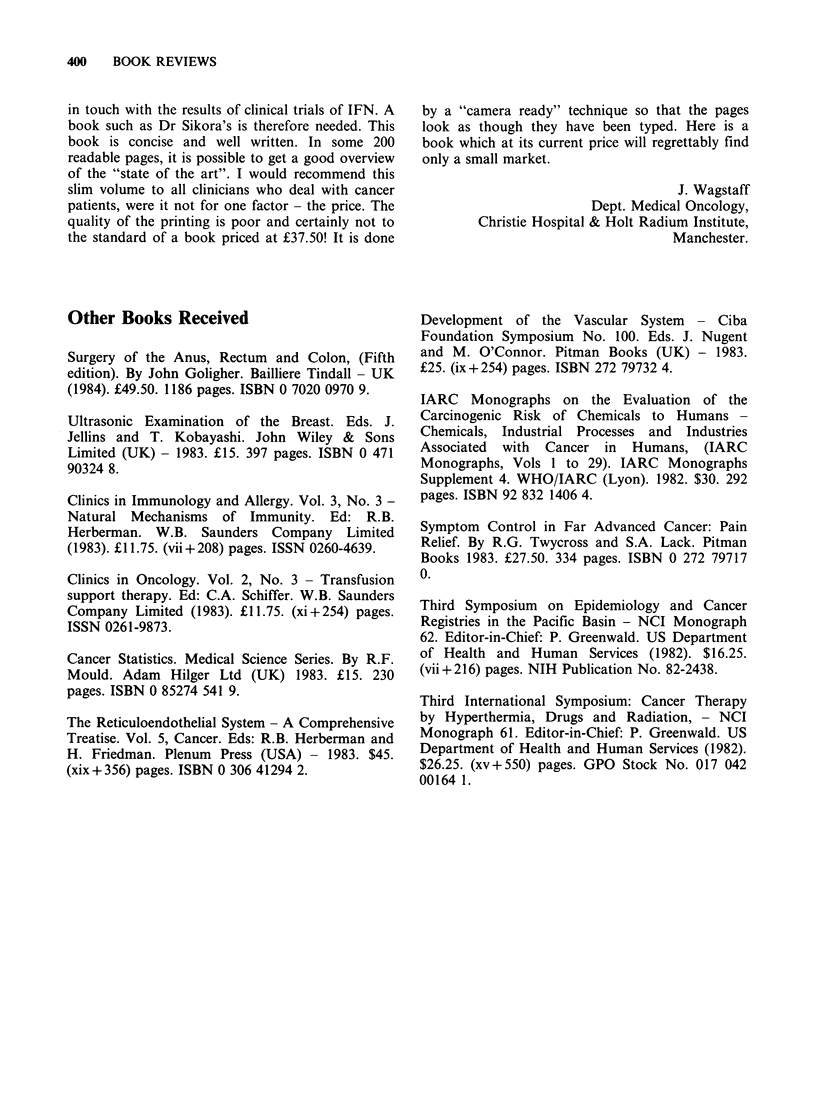# Interferon and cancer

**Published:** 1984-03

**Authors:** J. Wagstaff


*Interferon and cancer.* KAROL SIKORA, UK, Plenum Press, viii+215 pp, 1983, £37.50, 0 306 41379 5.

The interferons (IFN) were discovered over 25 years ago and the media have distorted their importance, as anticancer agents, beyond the fact. Many believe that IFN is a drug with proven efficacy against cancer and viral infections. Because of this, there cannot be a clinician who deals with cancer patients who has not, at some time, been asked about its availability. Yet, except for those of us who work in clinical oncology, few clinicians will be completely "au fait" with the results of clinical research into IFN. Their views will be largely prejudiced by their reading of the popular press. The application of modern molecular biological techniques to the area of IFN production means that for the first time, highly pure IFNs may be produced in virtually unlimited amounts. These substances are currently undergoing Phase I/II testing and will shortly become more widely available. It is therefore becoming increasingly important that clinicians who deal with cancer are in touch with the results of clinical trials of IFN. A book such as Dr Sikora's is therefore needed. This book is concise and well written. In some 200 readable pages, it is possible to get a good overview of the "state of the art". I would recommend this slim volume to all clinicians who deal with cancer patients, were it not for one factor – the price. The quality of the printing is poor and certainly not to the standard of a book priced at £37.50! It is done by a "camera ready" technique so that the pages look as though they have been typed. Here is a book which at its current price will regrettably find only a small market.

J. Wagstaff
Dept. Medical Oncology,
Christie Hospital & Holt Radium Institute,
Manchester.